# Dental Surgery

**Published:** 1885-07

**Authors:** 


### "DENTAL SURGERY."

In "Current News" for this month may be found an extract from *The New York Medical Record,* with the comments upon it of one of our associates. That journal is very poor reading for dentists, for it never misses an opportunity to depreciate and insult a class of men, a large proportion of whom have received the same medical training as its churlish editor. The extract is invested with a grace peculiarly its own, when it is remembered that this same editor is the Dr. Bliss of the Grant case. It is the name of Shrady that was signed to the sensational, almost hourly bulletins, that were a scandal in the eyes of so many medical men, and that have caused the making of such unpleasant comparisons between the management of the cases of Gen's Grant and Garfield. We do not know that any parallel can be drawn between the ability that Dr. Shrady exhibits in diagnosis and prognosis and his judgment concerning questions of professional ethics, but certainly, in the latter he does not show to advantage, whatever may be his fame in the former.

Of course every candid man who knows anything of the subject at all, knows that laws for the regulation of dental practice do not in any way interfere with medical practice or surgery. But the degree of M. D. is not a patent of universal accomplishment, and if we may judge of medical men by some of the articles that have lately appeared in *The Record*, common oral diseases would appear to be a subject of which they are most ignorant.

